# Challenges in modelling dynamic processes in realistic nanostructured materials at operating conditions

**DOI:** 10.1098/rsta.2022.0239

**Published:** 2023-07-10

**Authors:** Veronique Van Speybroeck

**Affiliations:** Center for Molecular Modeling, Ghent University, Technologiepark 46, 9052 Zwijnaarde, Belgium

**Keywords:** spatial disorder, machine learning potentials, kinetics, nanostructured materials, heterogeneous catalysis

## Abstract

The question is addressed in how far current modelling strategies are capable of modelling dynamic phenomena in realistic nanostructured materials at operating conditions. Nanostructured materials used in applications are far from perfect; they possess a broad range of heterogeneities in space and time extending over several orders of magnitude. Spatial heterogeneities from the subnanometre to the micrometre scale in crystal particles with a finite size and specific morphology, impact the material's dynamics. Furthermore, the material's functional behaviour is largely determined by the operating conditions. Currently, there exists a huge length–time scale gap between attainable theoretical length–time scales and experimentally relevant scales. Within this perspective, three key challenges are highlighted within the molecular modelling chain to bridge this length–time scale gap. Methods are needed that enable (i) building structural models for realistic crystal particles having mesoscale dimensions with isolated defects, correlated nanoregions, mesoporosity, internal and external surfaces; (ii) the evaluation of interatomic forces with quantum mechanical accuracy albeit at much lower computational cost than the currently used density functional theory methods and (iii) derivation of the kinetics of phenomena taking place in a multi-length–time scale window to obtain an overall view of the dynamics of the process.

This article is part of a discussion meeting issue ‘Supercomputing simulations of advanced materials’.

## 1. Introduction

Within this perspective, the question is addressed in how far current modelling strategies are able to model dynamic processes in realistic nanostructured materials at operating conditions. To set the scene, the used terminology is clearly defined. ‘Dynamic processes’ refer to any phenomenon where dynamic changes of the material or guest species interacting with the material are involved. This needs to be interpreted in a very broad way and encompasses for example catalytic reactions in nanoporous frameworks, phase transformations in framework materials under influence of external stimuli, adsorption in porous frameworks and many other cases. When discussing dynamic phenomena in nanostructured materials, it is important to realize that the material's dynamics is entangled with spatial heterogeneities present in the material. For this, the terminology spatio-temporal processes have recently been coined [1]. One illustrative example is the recent experimental findings in the field of metal–organic frameworks (MOFs) where it was shown that the dynamic response of flexible MOFs is largely impacted by the presence of defects and the crystal size of the material. Downsizing MOF crystals from the micro- to the mesoscale substantially suppresses their ability to morph between various phases [2–10]. This is only one example, but when carefully inspecting the literature many other examples can be found.

‘Realistic materials’ refer to materials as they are used in experimental set-ups, ‘operating conditions' relate to the assembly of external conditions under which the material shows the desired functional behaviour such as temperature, pressure, pH, moisture. Lastly ‘nanostructured materials’ refer to materials with tunable dimensions on the nanoscale. Such materials possess fundamentally different chemical and physical properties than bulk materials and show great potential within different application fields such as catalysis, sensing, light harvesting, energy conversions, to name only a few. The ultimate dream is to obtain absolute control in building structures at the atomic scale to obtain the desired functional behaviour at the macroscopic scale under the desired conditions. This is a very ambitious target both for theoreticians and experimentalists. Even if theoreticians would succeed in predicting how the ideal material would have to be built at the atomic scale to obtain the desired functional behaviour, experimentalists would have to be able to synthesize these nanometre-designed materials with atomic precision and to characterize them with the highest possible resolution. In the last decennia, modelling has matured substantially, evolving from explanatory modelling upon request of the experimentalist, to modelling in close synergy with experimental groups [11,12]. In my opinion, modelling has now reached a level where a synergistic modelling–experimental approach may lead to rational material design by using a constant feedback loop between modelling and experimental results. Within the last decades, the field of material's modelling has substantially evolved, thanks to major evolutions in available computing power and the development of innovative algorithms and theoretical methods. However, modelling has not yet reached the phase, to causally relate macroscopic behaviour with atomistic scale behaviour and to predict without experimental knowledge the outcome of a so-called thought experiment. The main reason for this is the discrepancy between experimentally relevant length and time scales and attainable theoretical length–time scales and overall the complexities present in realistic materials under operating conditions. Within this perspective, some key challenges are identified to further close the gap between a bottom-up atomistic modelling and top-down experimental approach within the field of modelling realistic nanostructured materials at operating conditions.

## 2. Spatial gradients, disparate time scales and complexities under operating conditions

**Figure 1.**
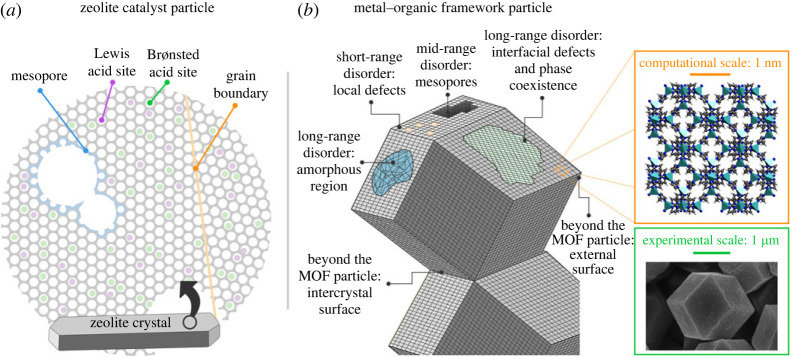
Spatial heterogeneities in (*a*) a zeolite particle and (*b*) a MOF particle. Adapted from [1,13,14] and with permission of Elsevier, Springer Nature and the Royal Society of Chemistry. (Online version in colour.)

Materials used in experimental set-ups have a finite size crystal size and a certain morphology. The size of the crystal particles may vary largely but usually extends from the nanometre to the micrometre scale. An illustration of a scanning electron microscope (SEM) image of ZIF-8 is shown in figure 1 [13]. Furthermore, realistic materials are not perfect and exhibit heterogeneities in space and in time, both at the crystal particle level but also beyond [15]. The latter refers to the way crystal particles are dispersed in supports or binders in order to apply the materials in industrial settings. Within the crystal particle, all kinds of spatial gradients may exist which may be either short-, mid- or long-ranged (figure 1) [16–18]. Examples of short-range defects could be missing linkers in MOF or the presence of extraframework aluminium species in zeolites [19,20]. Examples of the mid-range disorder are mesopores whereas long-range disorder could correspond to nanodomains or the inherent presence of external surfaces. Some illustrative visualization of spatial heterogeneities for a MOF particle and zeolite catalyst particle are shown figure 1. Previous examples refer to crystalline materials, yet today more exotic material phases are explored, such as liquid or glassy states within the MOF field [21].

Apart from spatial gradients, materials evolve in time, their evolution and function critically depend on the external conditions in which they are brought such as temperature, pressure, pH, presence of moisture. Properly taking into account these operating conditions within a modelling strategy is of utmost importance to give any viable prediction about the functional behaviour of the material. The last few years, a paradigm shift has taken place in computational modelling from static techniques where only a few (meta)stable states are modelled towards operando simulation methods that capture the functional behaviour under working conditions. Operando modelling can certainly not be achieved using only one single technique; instead, a range of models based on molecular dynamics (MD) methods, microkinetic models and machine learning algorithms are currently explored [22–24]. The inherent problem when modelling the dynamic response of the material is the inherent broad set of intrinsic time scales relevant for the dynamic evolution of the material ranging from the ps to the ms range. The shortest time scales are found in molecular motions and vibrations which occur on the (sub)picosecond time scale, whereas activated processes are not captured in this narrow time window. Within the field of heterogeneous catalysis, much research has been devoted to understand how the crystal morphology and size influence the transport and catalytic function. An overall integrative view on the entanglement between transport, kinetics and the way these are affected by the spatial heterogeneities is still an open question. The terminology spatio-temporal evolution of a material has been coined, referring to the fact that the dynamics of the material is affected by the material's spatial properties [1,25]. Looking from a more holistic perspective, it is important to realize that materials and processes have an intrinsic life cycle. This concept has been nicely documented in catalysis research and referred as the ‘birth, life and death’ of a catalytic solid [26,27]. Birth refers to the fact that a freshly prepared catalyst material first needs to be activated when loaded in the chemical reactor. During this activation phase, the material structurally changes to a point where it enters the active period of its life cycle. Even in the active period, catalytic solids show a heterogeneous spatio-temporal behaviour. Comparable with enzymes, which are found to ‘sleep and work’, catalyst may show a similar on–off behaviour [28–31]. Major efforts have been undertaken within the field of spectroscopy, imaging and characterization to follow the spatio-temporal evolution of the material. Operando spectroscopy was introduced around 2000 within the field of heterogeneous catalysis and has since then greatly evolved and allowed to spectroscopically follow how an operating catalyst is working [32–37]. Yet even today, it remains also for experimentalists a formidable challenge to follow on the fly the dynamic state of a material. A nice example is the stimuli-responsive behaviour observed for some MOFs, where a material shows bistable or multistable behaviour upon exposure to external stimuli such as temperature, pressure and gas adsorption [38]. Experimentally, framework flexibility can be recognized by monitoring the response of the material upon exposure to the external stimulus; however, to date it has proven impossible to follow the dynamics of the transition on the fly [25,39]. This is caused by the fact that the dynamic response is dictated by the occurrence of metastable states which are separated by energetic barriers and which are hard to assess experimentally. Major experimental efforts are currently undertaken to build *in situ* cells to unravel the spatio-temporal response of MOFs under operating conditions; however, to date the realistic time scales and the kinetic response of these soft porous frameworks are yet to be resolved [2,14,25,40].

## 3. Open questions and challenges in modelling dynamic processes in realistic nanostructured materials at operating conditions

Having introduced typical length, time scales and complexities within realistic materials, we can refer back to the initial question namely in how far we are capable of modelling dynamical processes in realistic materials under operating conditions. Obviously, the cited experimental dimensions both in space and time are much larger than the currently attainable length and time scales in modelling. We are thus today not yet able to model realistic materials under operating conditions and taking into account their full complexity. One of the greatest challenges within material's modelling is how to model operando functional behaviour of nanostructured materials within a similar spatio-temporal window as attainable experimentally. One could argue that computing power has massively expanded the last years, with the installation of very powerful high-performance computers. Very recently the first exascale computers have been taken in operation, which open a lot of perspectives for computationally very demanding disciplines, such as computational material's science. Further optimizations of accessible length/time scales can be achieved with the help of massive parallelization, extensive usage of graphical processing units (GPUs), dedicated software, hardware architectures and innovative methods as often done in biomolecular modelling [41–43]. However, as we will argue below, having access to such massive computing power alone is not sufficient to close the length–time scale gap between simulations and experiment. Fundamental methodological leaps forward are necessary to reconcile the macroscopic experimental observations with atomistic simulations. The length–time scale gap is not unique within the field of material's modelling but is inherently present in any field where one tries to bridge from the atomic scale to the macroscopic observable scales. Within the field of biomolecular modelling, many method developments have taken place to overcome the length/time scale gap. Some of these methodologies can be inspirational for the field of materials modelling; however, a straightforward extrapolation of methods of one field to the field of material's modelling is not possible due to particularities specific for the applications of interest.

**Figure 2.**
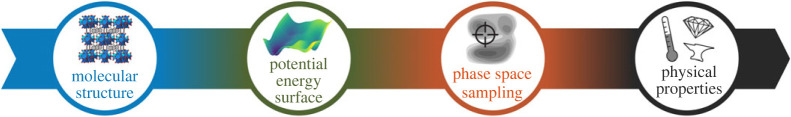
The sequence of a modelling exercise where one starts from a realistic atomic level representation for the material, determines the multidimensional potential energy surface (PES). This PES needs to be sampled in the interesting regions after which the target properties can be derived. (Online version in colour.)

To understand the main bottlenecks in bridging the length–time scale gap, one needs to carefully inspect all steps of a typical molecular modelling exercise. Figure 2 gives a schematic overview of the typical steps one needs to undertake when modelling a material. The sequence of a molecular modelling exercise starts with building a realistic atomistic representation for the material under consideration. In the next step, one needs a method to evaluate the forces between the atoms and obtain a reliable description of the potential energy surface (PES). It is important to realize that the PES is a highly dimensional function in terms of the internal coordinates. The desired thermodynamic and kinetic properties need to be derived in the last two steps by sampling the PES under the right conditions. Major methodological challenges exist at all levels of this modelling exercise as detailed below.

### (a) Challenge 1: building atomistic models for realistic materials

The first major challenge in modelling realistic materials consists in building atomistic models which are representative for the real material. Realistic atomistic models need to mimic as close as possible the material used under experimental conditions. Given the plethora of spatial heterogeneities present in realistic materials, this in itself is already a huge challenge. Most current material's modelling efforts adopt periodic boundary conditions and make use of a supercell representation with hundreds to maximum thousands of atoms, having dimensions of the order of nm. These dimensions are representative for models where the PES is modelled using quantum mechanical (QM)-based methods, when using force fields methods the accessible length scales increase as will be commented in the next challenge.

Atomistic material representations having dimensions in the nanoscale range, can only capture spatial gradients on the short range. If one aims to model materials capturing a more diverse set of spatial heterogeneities extending to longer length scales, one is confronted with the challenge how to build initial atomistic models for these spatially extended systems.

In the literature, a variety of attempts have been undertaken to capture short-range defects in nanostructured materials [44]. In principle, a brute force approach can be followed, where one starts from the perfect atomistic representation of the material and then systematically builds models where all theoretically possible defects are included. While such approach is valuable, the number of possibilities generated in such way grows very fast.

**Figure 3.**
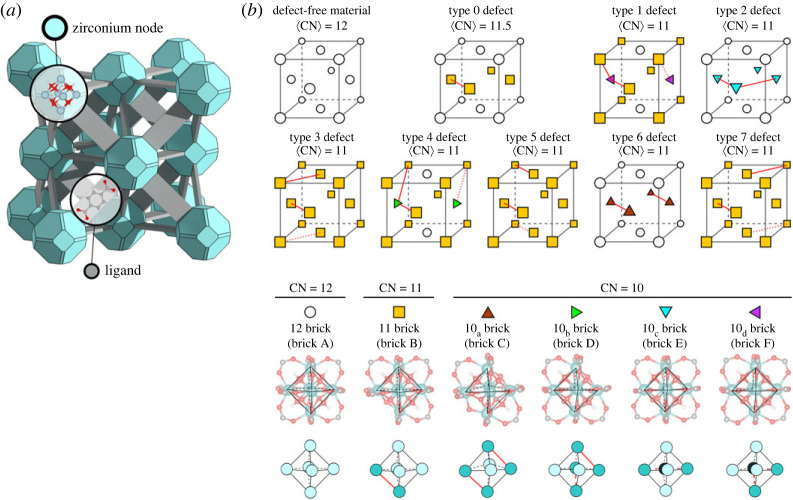
Visual representation of the UiO-66 material and its various defect structures. Reprinted from and adapted from [44] with permission of the American Chemical Society. (Online version in colour.)

This is hereafter illustrated for the UiO-66 MOF. The perfect material consists of 456 atoms per unit cell, contains four inorganic bricks inorganic Zr6(μ3-O)4(μ3-OH)4 bricks connected through ditopic organic ligands as shown in figure 3 [45,46]. In the defect-free material, each inorganic node is connected through 12 linkers. Experimentally, there is now consensus that the as-synthesized material always misses a certain degree of linkers [47]. Depending on the synthesis conditions, one can modulate the percentage of missing linkers. Based on these observations, the concept of defect engineering was introduced, where one tries to tune the properties of the material by meticulously controlling the number and type of defects [48–51].

When trying to construct molecular models for these missing-linker systems, one can start from the pristine material and remove any of the 24 linkers as they are all symmetrically equivalent [44,52]. When an additional linker needs to be removed, 23 possibilities exist, of which some of them are physical equivalent. As was shown by Rogge *et al*. [44], finally seven classes of defect structures can be created. When trying to remove even more linkers it is evident that the combinatorial space vastly expands and that it is not feasible to consider all possibilities. To bring systematics in the potentially plausible defect structures, one could work with defect classes, first investigate them in an uncorrelated way and then investigate in how far defects are correlated and give rise to collective phenomena. This would become relevant when a few defective structures coalescence to larger aggregated defective structures. At this moment, it is nearly impossible to build from scratch without experimental input realistic models for such defective materials. A tight connection is necessary with experimental spectroscopic characterization techniques to reveal as detailed as possible information on the defective structures. The earlier mentioned UiO-66 is a showcase example for which detailed spatially resolved measurements have been performed that yield information on the specific distribution of defects within the sample [53–55]. Many spectroscopic techniques are able to give information on the ensemble average defect concentration, for example, NMR, thermogravimetric analysis; however, no local defect arrangement is obtained. One can go a step further by using other bulk spectroscopic techniques like FTIR, NMR, EXAFs or total scattering X-ray diffraction methods; however, still one obtains as such ensemble average information on the defect arrangements. To provide informative input for building atomistic models, one needs to have access to spatially resolved measurements on how defect distributions are arranged within the sample. Particularly interesting are the advances made in high-resolution electron microscopy (HRTEM), a technique which in principle offers the possibility to directly observe individual defects in real space with atomic resolution [53]. However, one can certainly not use this technique in a standard set-up as the materials under consideration are beam sensitive, where the electron beam can easily damage structures. Recently, various HRTEM images have been developed and also successfully applied to the UiO-66, which gave detailed insights into the distribution and correlation of defects in the material [53]. From the spectroscopic characterization perspective, the field is in full development and also other techniques are explored such as scanning electron diffraction [54] and more general electron diffraction and microscopy imaging coupled with automated data processing [56]. Such measurements are very valuable for the modeller to construct in a targeted way atomistic models for realistic materials with spatial gradients varying from the nanoscale to the mesoscale range. To the best of our knowledge, these high-resolution experimental images have not yet resulted in realistic bottom-up atomistic mesoscale models. To further progress in this area, building algorithms have to be developed that allow to generate atomistic models in a systematic way, which matches high-resolution images obtained experimentally.

Previous observations relate to bulk materials which are modelled using periodic boundary conditions. In principle, realistic materials need to be modelled as finite-sized crystallites having sizes ranging from about 50 nm to the μm level. Simulations on finite-sized nanocrystallites are very scarce in the field of nanostructure materials [57]. Smaller metal particles have been simulated with density functional theory (DFT), but their sizes are much smaller in the order of 5 nm [58,59]. Within the MOF field, Keupp & Schmid [57] initiated for the first time finite-size simulations on nanocrystallites of DMOF-1(Zn) by setting up a crystallite model containing roughly 250 000 atoms, with explicit inclusion of the external surface. In their simulation, no periodic boundary conditions were used and a series of simulations were performed in a temperature ramp between 300 K and 500 K to observe the thermal opening, and constrained simulations were performed to observe the mechanical closing of the DMOF-1 system. Based on these simulations, important information was obtained on the nucleation mechanisms of transitions between various phases in soft porous crystals and how the surface-to-volume ratio affects the transition dynamics. Although the crystal sizes in the previous examples are still smaller than the experimental dimensions, the finite-sized crystal simulations are inspirational, to build more realistic models for crystal particles.

**Figure 4.**
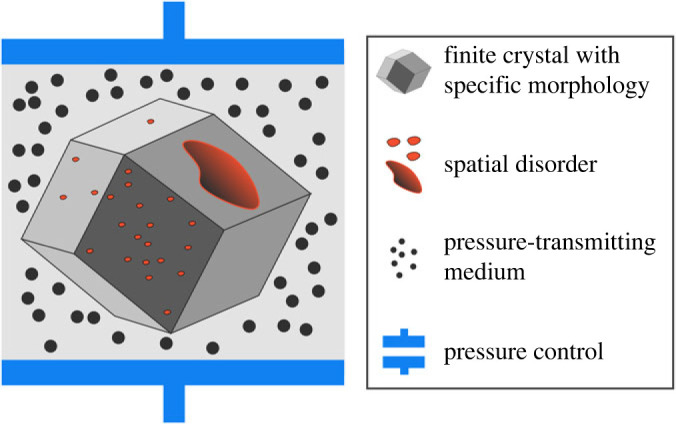
Schematic of a finite-sized crystal particle, with inherent spatial disorder and embedded in an external medium to exert pressure or other stimuli. Reprinted and adapted from [62]. (Online version in colour.)

To progress in this area, systematic methods need to be developed to build finite-sized particles with a certain size and morphology. This could be done using the atomistic Wulff construction method, where a three-dimensional surface is constructed by determining crystal faces with low surface energy [58,60,61]. To truly mimic experimental set-ups, finite-sized crystals with a specific morphology need to be embedded in an external medium which can exert in a more natural way an external pressure or simulate a heat bath, as schematically shown in figure 4. Several methodological advances are necessary to construct such finite-size embedded crystallites. The first crucial question is how to terminate the surfaces and how to model the particles of the external medium and specify their interaction with the external surface of the crystal. If the medium only has the function to exert an external pressure, as is for example the case in mercury porosimetry measurements [63,64], one would have to calibrate the potential to prevent the particles to enter the crystal. However, when one aims to model also possible reactive events or sorption within the material, explicit realistic interactions between the external surface and the particles of the external medium have to be considered. Another point of attention in modelling the finite-sized embedded crystallites, concerns the thermodynamic ensemble in which the system is modelled. One could embed the extended system in a periodic box and use the known thermodynamic ensemble algorithms. Ideally, the system becomes large enough and the simulations could be performed in the NVE ensemble where external stimuli such as temperature or pressure would be naturally imposed by the heat bath or the pressurizing medium. Extensive testing would be required, to determine the size and conditions necessary to obtain proper thermodynamic and statistical quantities. As an alternative strategy to mimic crystals in an external medium, one could make use of constant pressure temperature algorithms proposed for non-periodic systems, such as the Langevin Hull formalism, which relies on coupling the system with an external reservoir with fixed constant pressure (*P*), temperature (*T*) with an effective solvent viscosity (*h*) [65]. In any case, extensive testing would be necessary to determine in which set-up reliable structural, mechanical, thermal properties are obtained for real crystallites and give the correct thermodynamic properties.

Summarizing this first challenge, it is clear that major methodological advances are necessary to build more realistic atomistic models for nanostructured materials in the mesoscale range. Furthermore, a close interaction loop with experimentalists will be necessary to have access to spatially resolved information on the specific arrangements of defects at the atomistic level. For some benchmark materials, such information has recently become available and these materials are ideal cases for the modeller to initiate the development of algorithms which allow to mimic the high-resolution images. For modelling finite-sized crystals embedded in an external medium, modelling efforts would greatly benefit from close interactions with experimentalists to provide detailed information on surface termination and give detailed dependence of the material's properties in terms of the crystal size. Such information would be invaluable to benchmark future theoretical models in this area.

### (b) Challenge 2: efficient methods to describe the potential energy surface with quantum accuracy on larger length and time scales

Even when one would succeed in constructing representative atomistic models for the material at the mesoscale, one is confronted with the challenge on how to model the interactions between the atoms of these extended systems. The main reason for the discrepancy between theoretical and experimental attainable length scales, finds its roots in the method used to describe the PES. The PES is the central quantity in the description of any molecular problem as it describes the energy in terms of the internal coordinates of the system. Inherently the PES is a complex multidimensional function, which cannot be easily visualized. In principle, one should model the PES using QM-based methods, i.e. by solving the Schrödinger equation for the many-body electronic system. In most material's simulations, the nuclei are treated as classical particles and the dynamics of the electronic degrees of freedom is separated from the nuclear motion, within the so-called Born–Oppenheimer (BO) approximation. When the BO approximation is not valid, more advanced treatments have been proposed for which we refer to dedicated literature works [66–68]. Within the BO approximation, the dynamics of the nuclei is simply treated by considering classical particles moving on the underlying PES [69,70]. For most processes taking place within material physics, this is a good approximation; however, whenever light particles such as protons become important, nuclear quantum effects (NQEs) may become important [71]. This might especially be important for heterogeneous catalysis taking place in zeolites where proton hopping processes occur. Extensive testing is necessary to investigate in which temperature window such effects become important. Theoretical rigorous methods have been devised to account for NQEs, such as the path integral molecular dynamics (PIMD) approach, which relies on Feynman's path integral formulation of quantum mechanics [71–73]. Despite being conceptually very attractive, PIMD simulations are computationally very expensive and have scarcely been used within the field of modelling nanostructured materials or heterogeneous catalysis. However, for other systems clear evidence was given that NQEs may have a significant impact on the physico-chemical properties of systems containing light atoms [74,–76]. While being fundamentally very important, the problem of NQEs and how to address them is not extensively discussed in this perspective.

**Figure 5.**
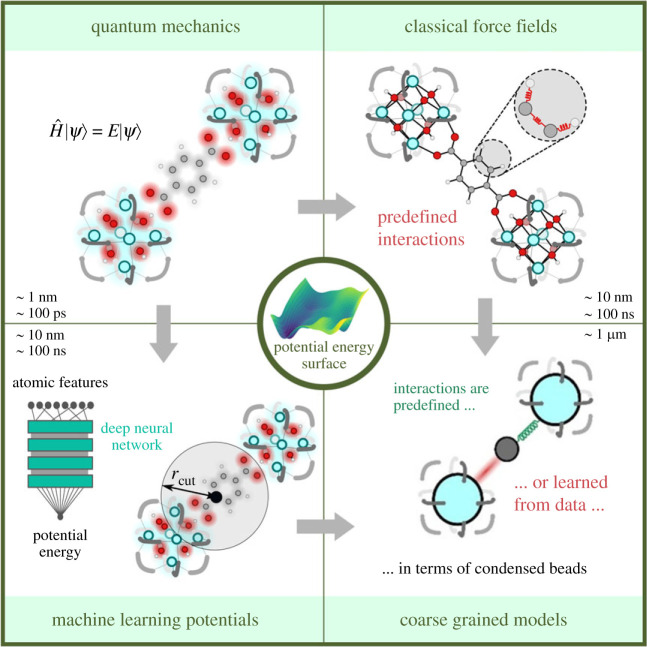
Overview of various theoretical methods to calculate the PES varying from quantum mechanics based methods and classical force field-based methods to machine learning potentials and coarse grained models. Typical accessible length and time scales are also indicated. Adapted from [1] with permission of Elsevier. (Online version in colour.)

Herein, focus is set on methods to describe the PES. Various approaches could be followed, as schematically shown in figure 5. Each method leads to a specific window of accessible length and time scales in the simulations. In the light of the initial question, namely how to model realistic materials under operating conditions, particular focus is given on methods where the electronic structure problem is modelled using QM-based methods. With these methods, the interactions are described from first principles and all sort of interactions and phenomena including those events where bonds are formed or broken can be accounted for. The upper left panel of figure 5 refers to QM-based methods [77]. Today, DFT has become the method of choice within chemistry and materials modelling, given its computationally efficiency versus accuracy. The accuracy of DFT methods critically depends on the exchange-correlation functional [78–81]. The particular choice of the energy functional is not always trivial and may be problematic for some cases such as the description of long-range dispersion interactions [82–86], which is for instance crucial to accurately determine the stability of intermediates in the pores of zeolites [87–91]. Also when interested in describing systems with strong electron correlations, DFT becomes problematic. As the focus within this contribution is on the quest how to model realistic materials at operating conditions, we do not comment in detail on the cases where DFT itself fails. The interested reader is referred to more dedicated works on this topic [92,–94]. In what follows the terminology ‘quantum accuracy’ will be used to refer to methods where the PES is modelled at an accuracy achievable with a certain QM-based methodology. Currently, for the systems under consideration in this paper—namely nanostructured materials—DFT is the method of choice for solving the electronic structure problem (vide supra). However, in principle, other many-body techniques, such as Hartree–Fock, post-Hartree–Fock or other many-body techniques may be used to solve electronic structure problem [66]. Every many-body technique has its own inherent level of accuracy compared with the exact solution and thus quantum accuracy relates to the accuracy achievable with a certain QM-based methodology. This terminology is used to make a clear distinction with methods where the electronic structure is not explicitly treated such as classical force field-based methods, where the interatomic interactions are modelled using simple analytical functions (vide infra).

The terminology quantum accuracy used here, should not be confused with the terminology ‘chemical accuracy’ or ‘quantum chemical accuracy’ which refers to methods that aim to obtain energies with an accuracy of 4 kJ mol^−1^ compared with exact benchmark values [95]. DFT with the current generation of functionals often does not reach these stringent criteria of chemical accuracy [91]. Complicated composite schemes, wavefunction-based methods or embedding schemes have been devised to target chemical accuracy in various application areas. Within the field of nanoporous materials seminal work has been performed by Sauer *et al.* [91]. Within the field of biocatalysis Thiel and co-workers [96] demonstrated that advanced wavefunction-based methods beyond DFT were necessary to achieve accurate reaction barriers. These are only two notable examples of strategies where an approach beyond DFT was necessary to pursue chemical accuracy. The problem with more accurate methods like coupled-cluster methods with single, double and perturbative triple excitations (CCSD(T)), which is seen as the gold-standard for accurate electronic structure calculations, is their problematic scaling behaviour, i.e. *ca* N^7^ for CCSD(T). Such scaling behaviour with system size prohibits their usage on lager systems, which is the main reason why various schemes have been devised to pursue chemical accuracy ranging from composite schemes or embedding schemes where part of the system is treated with very high accuracy and other parts are treated in a computationally more feasible scheme [66,97,100]. Interestingly, recently other strategies are being explored to pursue chemical accuracy, such as the method of Burke at al. where a machine learning strategy was proposed to connect DFT densities with coupled-cluster energies [101]. A complete discussion on the failure of DFT and methods aiming to reach chemical accuracy is beyond the scope of thispaper.

When referring back to the initial question namely how to model dynamic processes within realistic nanostructured materials, it is interesting to note that with DFT, we can nowadays model systems having *ca* thousands of atoms having dimensions in the nm scale. While this is already a huge achievement, these length scales are still substantially smaller than the experimental crystal sizes which reach to the mesoscale as elaborated in the previous section. Considering these limitations, a growing effort has been undertaken to simulate systems with larger dimensions.

In an attempt to increase length/time scales of realistic systems, classical force fields have been extensively used, where the interactions between the atoms are approximated by simple analytical functions, neglecting the quantum description of the electrons [102–105]. The analytical functions are parametrized to mimic either experimental data or to mimic features of smaller-sized systems on which accurate QM calculations can be performed. The main drawback of classical force fields is their lack in accuracy compared with QM methods, lack of transferability, e.g. force fields derived under certain thermodynamic conditions are not necessarily applicable in other operating windows, their inability to simulate bond formation/breakage, inaccuracies in describing host–guest interactions. With these force fields the attainable length scales can substantially be increased leading to models having dimensions having tens of nanometres. Within the field of biomolecular modelling much larger length/time windows have been accessed with the help of massive parallelization, extensive usage of GPUs, dedicated software, hardware architectures and innovative methods [41–43]. Parts of these concepts and ideas can be adapted and transferred to the field of nanostructured materials; however, as nanomaterials have a completely different building pattern with their own specificities, simply adapting methods is not sufficient; however, we can be greatly inspired by the field of biophysics [106,–112]. We recently pushed the limits of current simulations, by performing the first classical force field-based simulation on a flexible MOF containing a million numbers of atoms, by exploiting the massive parallelism of state-of-the-art GPUs using the OpenMM software package [62,113]. To illustrate that methods need to be adapted when going from one application to the other; it is interesting to note that also a new barostat had to implemented which allows anisotropic cell fluctuations which are important for materials which can change shape upon exposure to external stimuli [62].

Previous efforts, where optimal use is made of modern parallelization and classical force fields, are very valuable and show how one can push the limits of accessible length and time scales; however, ‘quantum accuracy’ is lost in such approach. To mitigate this deficiency, major efforts are currently undertaken to develop methods that maintain the quantum accuracy but come at a much lower computational cost. A very interesting evolution is the development of machine learning potentials (MLPs) for complex systems, where a numerical potential is derived using some (nonlinear) regression procedure, based on underlying QM training data [114–123]. With accurate MLPs it would in principle become possible to evaluate forces and energies much more efficiently compared with DFT while still maintaining the accuracy of the underlying DFT training data. In principle derivation of MLPs can also be performed based on underlying QM data which do not originate from DFT, provided the necessary training data can be generated. However as mentioned above, taking into account the computational efficiency, DFT is currently the method of choice to generate underlying QM data. Various mathematical MLP frameworks exist, which can be broadly categorized into kernel regression methods, which determine the interaction energy by comparing a given configuration to a set of reference configurations, or neural network potentials which directly determine a high dimensional representation of the PES based on thousands or even millions of parameters [117,124]. When training MLPs, necessary validations should be performed such as evaluating the mean absolute error (MAE) on the energies and forces on an independent test set different from the training data. Such validations are also necessary to ensure that no overfitting is taking place. For more detailed reports on validations of MLPs we refer to dedicated reviews on the topic[125,126].

So far the number of research reports on MLPs within the field of nanostructured materials is still relatively limited. One of the major drawbacks so far was the huge amount of training data necessary to train reliable MLPs for complex materials. Within the MOF field, Eckhoff and Behler, reported on the development of a neural network potential for MOF-5 in 2019 for the first time. They used a Behler–Parrinello neural network based on *ca* 20 000 references QM configurations [127]. Other notable examples can be found in the field of hybrid perovskites where an on-the-fly generated MLP was developed requiring substantially less training data [128]. Within the field of zeolites, Erlebach *et al*., recently, reported on the development of a neural network-based MLP using the SchNet architecture based on an underlying diverse database of zeolite structures which were optimized with DFT. Within the last effort, an active learning scheme was used to include also higher-energy transition states. The finally used underlying DFT-based dataset contained about 33 000 structures. As such the finally obtained MLP succeeds in recovering thermodynamic stabilities, vibrational properties, as well as reactive and non-reactive phase transformations [129]. These are only a few recent examples within the field of materials research which show the potential of MLPs for modelling nanostructured materials. The feasibility of MLP-based approaches will be determined by the number of necessary underlying QM data to train an accurate MLP. When the training dataset becomes too large, even MLPs become intractable to simulate materials where large portions of phase space need to be sampled. Examples of cases where larger portions of phase space become important are for example phase transformations of soft porous crystals with complex pathways involving a lot of internal degrees of freedom or catalytic reactions in nanoporous frameworks. Furthermore, when one aims to model defective structures reaching dimensions in the mesoscale range, the question is how many training data need to be taken up to obtain reliable MLPs for all of these defective structures. Would some parts of the MLP be transferable and how many additional QM data are necessary to obtain reliable representations for the mesoscale material? In what follows a few interesting avenues are mentioned, without the intention of being comprehensive, as the field of MLPs is in full exploration.

**Figure 6.**
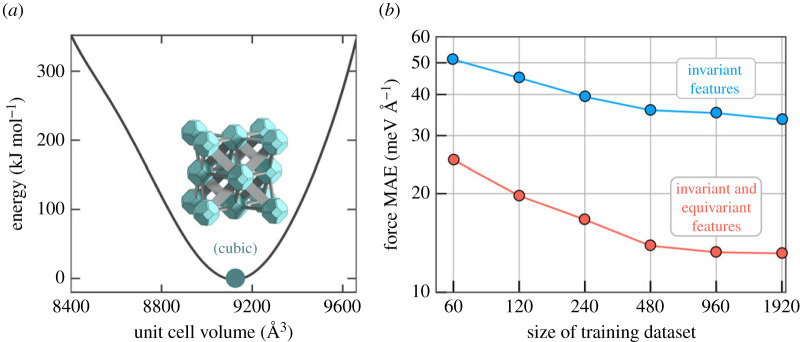
(*a*) Pressure versus volume curve for UiO-66(Zr) from which training data are extracted to train MLPs and (*b*) test error as a function of the size of the training dataset with rotationally invariant (blue, upper curve) and rotationally equivariant models (red, lower curve). (Online version in colour.)

First of all, when interested in training MLPs for complex materials, one needs to resort to mathematical frameworks that inherently need less training data. One notable example is the neural equivariant interatomic potential (NequIP) framework, which is message passing neural network that achieves remarkable data efficiency due to its use of rotationally equivariant features in the characterization of atomic environments [130]. Also, other frameworks have recently become available [131]. Figure 6 shows some of our recently obtained data for the UiO-66, showing that with NequiP, only a few hundreds of reference configurations are necessary to obtain mean average of forces below 20 meV Å^−1^, which is regarded as highly accurate in the field [132].

Second, in an attempt to reduce the number of necessary DFT input structures to the greatest extent while maintaining the necessary accuracy, innovative algorithms are necessary to select those structures that yield as much as possible new information to the training data. Various reports use DFT data obtained from underlying MD simulations, which are obtained from higher temperature simulations to enforce larger deviations around the equilibrium structure. In terms of reducing the number of expensive DFT calculations, this might not be the best strategy as subsequent structures from MD simulations are highly correlated. In this respect, it would be better to resort to active learning schemes where one starts from a restricted number of input DFT data and where one adds additional input structures on-the-fly whenever unknown regions of phase space are explored. The idea is to employ an iterative procedure in which the MLP itself is used to generate the training data. In each iteration, the MLP is used to sample the phase space until it encounters yet-unknown environments, which are then re-evaluated at the DFT level and again learned by the MLP. As such systematically better MLPs are generated by mingling in new DFT structures on the fly. The concept of active learning has not systematically been explored for nanostructured materials. To develop optimal strategies for generating in an efficient way training data for complex materials and activated dynamic processes, it will be necessary to optimally exploit the strength of enhanced sampling protocols. A vast literature on the topic has been developed but its connection to the field of MLPs and how MLPs can be used to perform part of the sampling is yet largely to be explored [133,134].

When referring back to the initial question on how to simulate realistic materials, having various degrees of spatial disorder reaching to the mesoscale, a major question concerns how to select defective structures on the nanoscale which can represent disorder in mesoscale systems. As MLPs strictly spoken represent short-range interactions, efficient algorithms have to be developed to detect representative structures on the nanoscale which are diverse enough to represent mesoscale disorder. Here, it would be necessary to develop systematic algorithms which allow to detect structurally diverse nanoscale regions in mesoscaled systems based on structural descriptors and atomic feature representations. Such algorithms could be compared with scanning algorithms where one scans the mesoscaled systems with a magnifying glass to detect nanoscaled portions that are structurally different from already included environments. Only when the nanoscaled systems are substantially diverse from already included environments in the dataset they could be calculated with DFT and added to the training set. One point of attention when modelling mesoscaled systems is how to represent long-range interactions [135,136]. In principle, MLPs are able to represent short-ranged interactions and testing would be necessary to investigate in how far MLPs need to be complemented with physical models to describe interactions in extended defect structures such as mesopores. Treatment of long-range interactions is in full development within the MLP community, various schemes have been proposed [117,123,136,138].

Previous paragraphs mainly comment on the development of MLPs for empty frameworks. Referring back to the initial question on whether we are capable of describing dynamic processes in realistic nanostructured materials at operating conditions, strategies need to be developed where also guest molecules are included in the materials which can also undergo chemical transformations. In principle there are no fundamental problems in training MLPs for reactive events; however, the main question is how to obtain underlying representative training data. The last decade major advances have taken place in studying chemical conversions at operating conditions using enhanced sampling molecular dynamics (ESMD) simulations where the forces are evaluated using DFT [139–144]. As explained earlier, simply performing DFT-based enhanced sampling MD simulations to start generating an underlying training dataset, would be very inefficient. More innovative algorithms will have to develop which combine concepts from active learning, enhanced sampling to train in a feasible way MLPs for reactive events [145]. A related question concerns in how far the obtained MLPs are in some sense transferable. For example, in how far are MLPs trained for a specific chemical reaction in one zeolite transferable to other zeolite frameworks? Suppose it would become possible to develop accurate MLPs for reactive events in nanoporous frameworks, a lot of possibilities to extend our insight on chemical reactivity would be opened. One could systematically investigate competitive pathways under various conditions include the effect of guest species and operating conditions in a moresystematic way.

Finally, when referring back to figure 5, with possible methods for describing the PES, the right bottom part refers to coarse grained (CG) methods. In this case, one abandons the atomic scale resolution of the system and groups the atoms in so-called CG beads, which interact with each other through an effective potential [106,146]. With CG methods the number of degrees of freedom is drastically reduced and one can bridge orders of magnitude in accessible length and time scales. However, with CG methods one loses atomic resolution and those methods cannot be classified as methods that describe the PES with quantum accuracy. For this reason, no extensive elaborations are given on this class of methods. On a side note, it is remarkable that so far CG methods are not extensively explored within the field of nanostructured materials, only a few examples of CG methods are reported [147,148]. This is in stark contrast to the field of biomolecular modelling, where extensive research has been performed on CG methods [106–108,149].

Summarizing this second challenge on methods to model the PES with quantum accuracy on longer length and time scales, it has become clear that one cannot resort solely to DFT-based methods. The field of MLPs is in vast expansion and offers the potential to extend accessible length and time scales with orders of magnitude while keeping the quantum accuracy of the underlying DFT data. However, major methodological hurdles need to be overcome to integrate MLPs as a reliable tool within the field of nanostructured materials, which relate to the efficient construction of an underlying DFT-based training data, efficient methods to explore unknown regions of phase space, how to incorporate in a founded way long-range interactions, how to incorporate guest species in the framework and simulate their reactive events at operating conditions. Lastly, whereas MLPs are very promising they are still not as efficient as classical force field-based methods and efficient implementations in massively parallelized frameworks will be necessary. Furthermore, other strategies such as hybrid approaches combining MLPs with classical force field descriptions might also be explored to further extend the accessible spatio-temporal window [118,138].

### (c) Challenge 3: integrative models for dynamic phenomena bridging various time scales

The third challenge deals with how to describe dynamic phenomena taking place in a broad range of time scales and connects with steps 3 and 4 of the molecular modelling exercise (figure 2). Suppose it would be possible to build realistic atomic representations of realistic materials and to describe the interactions between the particles with quantum accuracy for these extended models, then the next step consists in devising methods that allow to extract thermodynamic, kinetic properties and material's characteristics that connect with macroscopic observations. The exact approach to be followed for deriving these quantities largely depends on the property of interest. In any case, it is important to make the correct bridge between microscopic samples of the system and macroscopic quantities via the toolbox of statistical mechanics. For more information on how to derive macroscopic properties, we refer the reader to dedicated textbooks on the topic [150,151].

Herein we specifically focus on how to obtain kinetic information for various dynamic phenomena taking place in nanomaterials and also emphasize the challenges involved when dealing with systems where a broad set of (disparate) time scales are involved. This is certainly the case for most phenomena taking place in nanostructured materials, where time scales vary from the ps range that are typical for molecular motions to much longer time scales representative for chemical or physical transformations [152,153].

**Figure 7.**
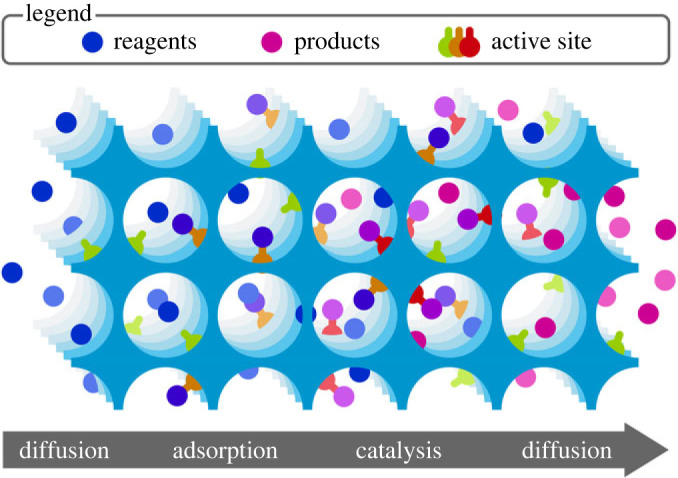
Schematic of a heterogeneously catalysed reaction, with various steps namely diffusion, adsorption of reactants, catalysis between reactive species and desorption and diffusion. Reprinted with permission from [139] with permission of Elsevier. (Online version in colour.)

A showcase example is encountered for a catalytic reaction taking place in a heterogeneous catalyst, as schematically visualized in figure 7. It consists of following steps: the reactants need to diffuse into the pores, undergo intracrystalline diffusivity to reach the active sites where they can react and lastly when the reaction has taken place, they need to desorb and be released from the catalyst. Each of the mentioned steps is characterized by vastly different time and length scales. Furthermore, transport phenomena are very dependent on imperfections within the crystal. As an example, it was found for real zeolites that the transport rates over long distances rather reflect the influence of the surface and internal barriers rather than diffusion through an ideal crystal [154,–160].

The problem of intertwined dynamic phenomena, which are dependent on spatial heterogeneities, is not unique for catalysis. Also within the field of sorption and sensing materials, guest species need to diffuse through the material, but might also undergo at some places in the material specific interactions with specific sites in the material or experience internal surface barriers due to spatial heterogeneities. A major question concerns how to model these phenomena in an integrative way and is it possible to derive kinetics for the overall process which is inherently of multiscale nature?

In what follows, some challenges in this area are illustrated for the case of catalytic conversions within nanoporous frameworks. However, various concepts are much more generally applicable for dynamic processes in nanostructured materials where phenomena of transport, specific host–guest interactions and activated processes are involved. The interested reader is also pointed towards some key papers and recent contributions on multiscale computational modelling within the field of catalysis [24,161,162].

**Figure 8.**
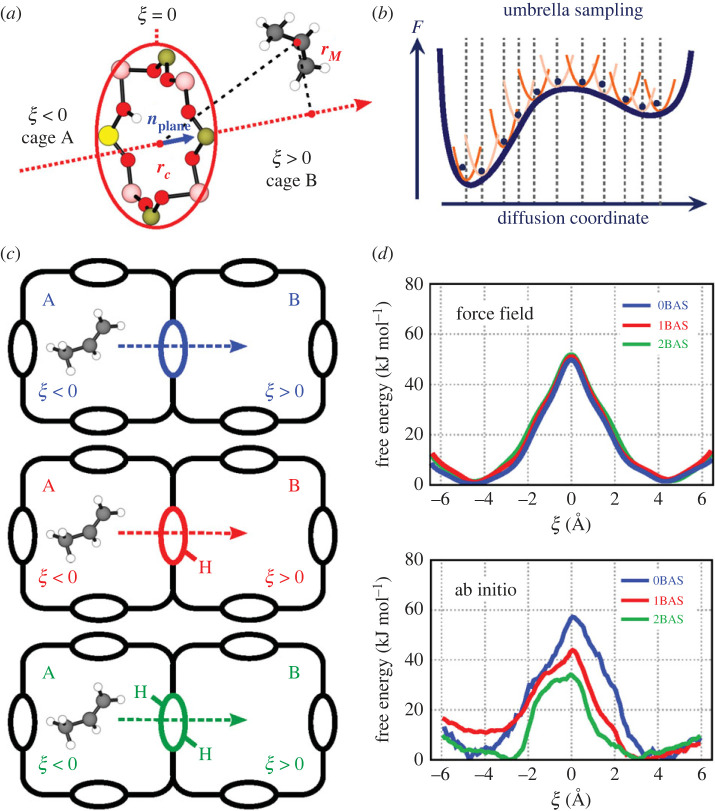
Illustration of enhanced sampling molecular dynamics methods to study hindered diffusion in zeolites. (*a*) Definition of the collective variable for propene diffusion through the 8-ring of H-SAPO-34, (*b*) schematic illustration of the umbrella sampling method to construct the free energy profile along the collective variable, (*c*) three molecular models for H-SAPO-34 with a varying number of Brønsted acidic sites in the 8-membered ring and (*d*) free energy profiles at 600 K constructed with a force field and using DFT-based methods. Figures adapted from [166,167] with permission of the American Chemical Society and Wiley. (Online version in colour.)

The problem with current modelling strategies in this area is the substantial difference in length and time scales involved for diffusion, reactive phenomena, etc. Diffusion typically takes place over longer distances (10–100 nm) and longer time scales, whereas reaction dynamics takes place in a more local environment. So far there is an inconsistency in the methods used to model transport phenomena, adsorption and reactive events. Transport phenomena are mostly modelled using classical force field simulations, given the extensive length and time scales involved [156,163,164]. Activated processes where chemical bonds are broken or formed need to be modelled using QM-based methods [142,165]. Despite the current modus operandi to model diffusion with classical force field-based methods, care needs to be taken with this approach as transport phenomena may be seriously affected by specific host–guest interactions. We recently discovered that interchange hopping of olefins in H-SAPO-34 is facilitated by the presence of Brønsted protons due to favourable π–H interactions with olefins, which cannot be captured with classical force field simulations (figure 8) [166,167].

A first step towards building an integrative kinetic model for processes characterized by disparate time scales such as diffusion and kinetics is to consistently model all steps in the catalytic process at the same level of theory and derive in a consistent way kinetic data for every elementary step. For catalytic conversions, this would boil down to studying external surface resistance, intracrystalline diffusion, adsorption and kinetics at the same level of theory. In what follows some comments are made for each of these individual contributions and open questions are formulated.

#### (i) Kinetics of activated processes

Activated processes have for a long time been modelled using so-called static approaches, where only a few points on the PES are considered from which the kinetics is derived via transition state theory (TST) or some straightforward extensions [142]. Specifically for a catalytic reactions, this leads to modelling states corresponding to the adsorbed states, transition states and products. Such approach has for a long time been the method of choice given its favourable computational cost, as only a few points on the PES have to be calculated. The extrapolation from ground state energies at absolute zero to thermodynamic properties at elevated temperatures is done via thermal corrections based on the normal coordinates evaluated around (meta)stable states. While for a long time, this was the method of choice for deriving kinetics of elementary processes, this harmonic oscillator approximation is a very crude approximation. Many examples are now available showing that such approach falls short for modelling reactive processes at operating conditions where a lot of complexity is involved such as strong anharmonic behaviour, dynamic host–guest interactions, dynamic evolution of active sites, participation of surrounding water to the reactive environment, etc [168–173]. The last couple of years, more efforts have been undertaken to model reactive phenomena at operating conditions. One method to achieve this goal is to make use of first principle MD simulations, where the dynamics of the system is followed in time via numerical integration of the Newtonian equations of motions in a thermodynamic ensemble representative for the governing operating conditions. When used in combination with enhanced sampling techniques, one can model activated processes taking into account the full dynamics of the system [174–177]. The main drawback of this approach is the computational expense, as each energy evaluation during the MD simulation is performed using DFT calculations. For example, a 100 ps MD run with a time step of 0.5 fs, requires 200 000 DFT energy calculations. When simulating chemical reactions, via an enhanced sampling technique such as umbrella sampling, metadynamics or another methodology, one quickly needs 500–1000 ps simulation time. These numbers clearly illustrate that a straightforward application of such MD-based methodologies with underlying evaluation of energies and forces based on DFT is not feasible as a standard tool in computational catalysis. To allow a more broad application, access is needed to methods which allow the evaluation of the PES at much lower computational cost than DFT, while providing the same accuracy. The latter point connects with challenge 2. Ideally, robust MLPs could be derived for chemical transformations and in general for activated processes which would allow to simulate in a more straightforward way these processes using ESMD methods. Having access to computationally cheaper methods with quantum accuracy, would also allow to achieve better sampling of the overall process and to derive in a more rigorous way the kinetics of the process. In current ESMD simulations, one recovers the free energy surface along a limited number of collective variables (CVs), which are chosen to steer the system towards less probable regions corresponding to activated events [178]. Such methodologies allow to obtain in a robust way differences in free energies; however, the extraction from kinetic information from such simulations is less trivial [140]. One particular point of attention is the dependence of the free energy on the chosen CVs [140,179,180]. Various methods have been proposed to derive the kinetics, which are independent on the particular choice of CVs.

To derive the kinetics from the simulations, one still relies on some variant of TST [140,181,183]. In principle, the most robust way to obtain the kinetic rate constant for an elementary reaction step would be the use of the Bennett–Chandler approach, where barrier recrossings are accounted for through the reactive flux formalism [184–186]. However, to achieve this, a large number of extra unbiased simulations need to be started atop of the transition state and monitored whether they end up in the reactant or product basin to assess in how far barrier recrossings take place. When using DFT-based energy evaluations, such direct kinetic evaluations were so far not feasible due to the computational expense. We recently performed a proof-of-concept study where a MLP was derived for a proton hopping event and where the kinetic rate constants were determined by directly applying the Bennett–Chandler approach [187]. With the availability of computationally cheaper methods for evaluating the PES with ‘quantum accuracy’, such approach would become feasible and it would be possible to derive in a more rigorous way the kinetics of activated processes.

To circumvent the problem with the choice of CVs, one could also resort to methods which do not necessitate an *a priori* definition of CVs. Within this class, transition path sampling (TPS) methods, where an ensemble of transition paths is created on the basis of an initial trajectory, is particularly promising [188–190]. It could also lead to the discovery of new transition paths. So far, TPS methods are hardly used in the field of nanostructured materials, as they are computationally expensive when used in combination with expensive DFT-based energy evaluations. Having access to methods that allow to evaluate the PES in a computationally more cheaper way would open a window of opportunity to apply TPS-based methods or variants like transition interface sampling. In such approach a reaction rate could be determined from the dynamics of the generated ensemble of transition paths [191–194].

#### (ii) Adsorption

The proper description of adsorption at operating conditions within nanoporous frameworks remains still very challenging [91]. Static approaches do not always predict the correct nature of the intermediates at operating conditions. One example for which much research has been performed to reveal the true nature of the intermediates at elevated temperatures is the adsorbed states of alkenes within Brønsted acidic zeolites. At higher temperatures, entropy contributions become more important, which promotes species which are more loosely connected with the framework such as physisorbed π-complexes or carbenium ions [170,171]. There is a very rich literature on the topic, as many factors contribute to the stability of the species such as the electronic level of theory to describe the host–guest interactions and the method to account for the entropy contributions [91,195,196].

MD methods are tractable for deriving adsorbed states as they can account for operating conditions. In principle, one can calculate adsorption enthalpies by taking ensemble averages of the internal energy and correct them by the necessary thermal corrections [87]. However, such approach is hampered by the limitations in accessible time scales when DFT-based methodologies are used. Furthermore, adsorption is very critically dependent on the level of theory used to describe the host–guest interactions and thus in this area DFT on itself is often not sufficiently accurate. Intelligent algorithms have been proposed, where one starts from a lower level of theory, i.e. DFT-based methodology, and then corrects the energies via thermodynamic perturbation and a selected number of energy evaluations at very expensive levels of theory such as the random phase approximation (RPA) [90,197]. Here concepts of Δ-machine learning are applied. The approach is very valuable but has its limitations as the phase space visited by the two levels of theory involved need to be similar. Seminal work has been performed by Sauer and co-workers over the years, where advanced hybrid schemes were proposed that aim to obtain chemical accurate adsorption enthalpies, i.e. within 4 kJ mol^−1^ from accurate experimental data [198,199]. To achieve this, complex calculation schemes are used where DFT energies for the full periodic cell are corrected by wavefunction-based methods that also include in a systematic way for electron correlation and second vibrational contributions are corrected with anharmonic corrections [169,200]. Whereas these methods are very valuable, they cannot be used in a routine way, due to the computational expense of the computational scheme. It would be interesting to have access to methods that combine the advantages of MD methods with proper configurational sampling with the accuracy of correlated electronic structure methods.

Apart from MD-based methods, adsorption and the influence of a varying number of particles have been widely studied using grand canonical Monte Carlo simulations [201]. Within such approach, typically a millions of energy evaluations are necessary, which is absolutely impossible to perform with DFT and hence classical force fields are mostly used in current modelling efforts in this area [202–204]. However, in some cases, explicit interactions occur with guest sites in the material and an approach beyond classical force fields is necessary. We recently proposed a more efficient algorithm based on importance sampling to select those structures that determine the behaviour in the low-pressure regime [205]. By adding a bias potential originating from a lower level of theory and only retaining those structures having sufficiently high probability to contribute to the property of interest, relevant structures for QM data generation can be selected. From this one could try to evaluate Henry coefficients and adsorption enthalpies which are similar to experimental data in the low-pressure regime with quantum accuracy. When interested in calculating the full isotherm with quantum accuracy, one needs to resort to other methodologies, where energies can be evaluated with quantum accuracy at lower computational cost. This is so far an open challenge and further development of algorithms that allow to determine adsorption isotherms with quantum accuracy would be very valuable [206].

#### (iii) Intracrystalline diffusion and external surface resistance

So far intracrystalline diffusion has been described mainly using force field-based methods; however, we proposed recently a methodology based on enhanced sampling in combination with DFT to derive free energy profiles for hindered diffusion [167]. This leads to free energies for the hopping process at realistic working temperatures and zeolite loadings from which diffusion constants can be derived [166]. This method has a lot of potential, however, similar to for deriving kinetics (vide supra), it is computationally very expensive [167]. Various umbrella samplings are performed along a CV describing the diffusion path (figure 8). It would be interesting to explore this methodology for other frameworks, where more complex CVs are necessary and ideally it could be coupled to cheaper methods to evaluate the PES.

External surface resistance is an essential contribution when aiming to construct integrative kinetic models for the kinetics and transport. Various techniques have been proposed in the literature to surface resistance from atomic simulations, which either rely on non-equilibrium MD or equilibrium MD and which allow to calculate the molar flux or diffusivity through the surface [156,158,207,210]. Ideally, these could be combined in combination with realistic models for the material, although such simulations will lead to very large simulation set-ups which need to be simulated for very long times. This is currently completely beyond reach for any DFT-based method. Hopefully, when having access to computationally more feasible methods to evaluate the PES with quantum accuracy, some progress could be made in this area.

Previous sections clearly reveal that many challenges are ahead within each of the separate contributions of an overall kinetic description of a multi-time scale problem. Suppose in the ideal scenario, it would become possible to evaluate each individual step sketched above at the same level of theory having quantum accuracy, it still remains a puzzle how to reconcile all steps and build on overall kinetic model. One can be inspired by the chemical kinetics of complex reaction networks rooted in chemical engineering and mathematics; however, it would be necessary to also integrate molecular-level concepts in these formalisms [211–213]. Recently, some studies appeared where models are constructed that account for hindered diffusion, surface barriers and kinetics starting from a top-down model and which are connected with measurements of temporal analysis of products (TAP) [214]. TAP measurements are unique in the sense that they can capture phenomena such as diffusion, chemical reactions but also contributions from surface barriers [214]. Ideally, bottom-up atomistic models can be reconciled with such top-down experimental approaches. With respect to experimental validation, it needs to be mentioned that the direct comparison of transport and kinetic data with experimental data entails a challenge on its own. Particularly for transport phenomena, seminal contributions appeared by Karger and co-workers [163,215,216]. Various techniques are available for obtaining information on transport phenomena such as quasielastic neutron scattering measurements to retrieve information on intracrystalline diffusion in submicron crystals [217], PFG-NMR measurements on large crystals to extract intracrystalline self-diffusivities [163,216,218,219] and TAP measurements were also other levels of complexities such as the surface barrier resistances or inhomogeneities in crystallite sizes are accounted for [220,221]. Given these complexities from experimental point of view and the necessary proper interpretation of the data, future modelling efforts are best performed in close synergy with experts in the various experimental techniques.

Summarizing this third challenge, it has become clear that deriving kinetics consistently for all steps involved in a multi-length–time scale phenomenon is very challenging. Current model strategies use different non-comparable levels of theory for the description of the interatomic interactions for each of the involved steps. Phenomena operative in larger spatio-temporal windows are described using classical force fields, whereas more local reactive events are modelled using QM-based methods. Having access to methodologies that enable a description of the PES with quantum accuracy at much lower computational cost (Challenge 2), would open a window of opportunity to derive in a consistent way the various steps of a multi-length–time scale process. The development of MLPs for the various steps might be an important element for building integrative kinetic models where a broad range of time scales are involved. However, also fundamental investigations will be necessary to mathematically construct overall consistent kinetic models for the process and to bridge from the atomic to the macroscopic scale.

## 4. Summarizing outlook

Within this perspective, the question was addressed in how far we are able to model dynamic phenomena in realistic nanostructured materials at operating conditions. Previous considerations made clear that despite huge advances in materials modelling, we have not yet reached the stage where theoretical modelling starting from a bottom-up atomistic approach can be reconciled with a top-down experimental approach. The typical length and time scales involved in both approaches are substantially different. Closing the gap between theory and experiment in this field, would necessitate the development of systematic algorithms to build mesoscopic models which are representative for experimentally used materials. A close interaction loop with the experimental community on imaging, spectroscopy and characterization will be of utmost importance to make progress in this direction. If modellers would succeed in building mesoscopic models, accurate methods need to be developed to describe the interactions with quantum accuracy albeit with much lower computational cost than currently applied DFT methods. The field of MLPs is in full development, however, its extension to the field of complex materials under consideration here and working in a variety of complex operating conditions, poses serious challenges and will require innovative theoretical, mathematical and computer models. Finally, when interested in describing the kinetics of dynamic phenomena taking place in realistic nanostructured materials, which are characterized by a broad range of time scales, methods need to be developed to consistently model each of the elementary steps at the same level of theory for the interatomic interactions. The information of each of these individual steps could then be combined in an integrative kinetic model, where each elementary step is modelled with quantum accuracy, leading to a bottom-up model that allows to bridge from the atomistic scale to the macroscopic scale relevant for experimental conditions. Summarizing, modelling dynamics processes in realistic nanostructured materials at operating conditions is very ambitious. Major steps forward may be achieved from collaborative efforts from theoreticians with various backgrounds and synergistic investigations with expert experimentalists.

## Data Availability

This article has no additional data.
